# Purification and Electron Transfer from Soluble c-Type Cytochrome TorC to TorA for Trimethylamine N-Oxide Reduction

**DOI:** 10.3390/ijms252413331

**Published:** 2024-12-12

**Authors:** Alka Panwar, Berta M. Martins, Frederik Sommer, Michael Schroda, Holger Dobbek, Chantal Iobbi-Nivol, Cécile Jourlin-Castelli, Silke Leimkühler

**Affiliations:** 1 Department of Molecular Enzymology, Institute of Biochemistry and Biology, University of Potsdam, Karl-Liebknecht Str. 24-25, 14476 Potsdam, Germany; panwaralka2016@gmail.com; 2 Department of Biology, Humboldt-Universität zu Berlin, Unter den Linden, 10999 Berlin, Germany; berta.martins@hu-berlin.de (B.M.M.); holger.dobbek@biologie.hu-berlin.de (H.D.); 3 Molekulare Biotechnologie & Systembiologie, RPTU Kaiserslautern-Landau, Paul-Ehrlich Straße 23, 67663 Kaiserslautern, Germany; frsommer@rptu.de (F.S.); m.schroda@rptu.de (M.S.); 4 CNRS, BIP, Aix-Marseille University, 13005 Marseille, France; iobbi@imm.cnrs.fr (C.I.-N.); jourlin@imm.cnrs.fr (C.J.-C.)

**Keywords:** molybdoenzyme, TorA, TorC, c-type cytochrome, TMAO reductase, Alphafold, crosslinking, electron transfer, X-ray structure

## Abstract

The enterobacterium *Escherichia coli* present in the human gut can reduce trimethylamine N-oxide (TMAO) to trimethylamine during anaerobic respiration. The TMAO reductase TorA is a monomeric, bis-molybdopterin guanine dinucleotide (bis-MGD) cofactor-containing enzyme that belongs to the dimethyl sulfoxide reductase family of molybdoenzymes. TorA is anchored to the membrane via TorC, a pentahemic *c*-type cytochrome which receives the electrons from the menaquinol pool. Here, we designed an expression system for the production of a stable soluble form of multiheme-containing TorC, providing, for the first time, the purification of a soluble pentahemic cytochrome-*c* from *E. coli*. Our focus was to investigate the interaction between TorA and soluble TorC to establish the electron transfer pathway. We solved the X-ray structure of *E. coli* TorA and performed chemical crosslinking of TorA and TorC. Another goal was to establish an activity assay that used the physiological electron transfer pathway instead of the commonly used unphysiological electron donors methylviologen or benzylviologen. An AlphaFold model including the crosslinking sites provided insights into the electron transfer between TorC_C_ and the active site of TorA.

## 1. Introduction

*Escherichia coli* is a facultative anaerobic bacterium that grows under different environmental conditions. It utilizes several alternative electron acceptors such as nitrate, dimethylsulfoxide (DMSO), and trimethylamine N-oxide (TMAO), particularly under anaerobic conditions. The TMAO respiratory complex in *E. coli* is encoded by the *torCAD* operon, and the expression of this operon is controlled by the TorS/R regulatory system, which is regulated by TMAO availability [1,2]. The presence of TMAO in the cells is detected by TorT, which activates the histidine kinase TorS [1]. Consequently, TorS autophosphorylates and transphosphorylates the response regulator TorR [2]. The phosphorylated TorR binds to the *tor* operon promoter, activating its expression [3]. TMAO reduction involves a membrane-associated pentahemic *c*-type cytochrome TorC and a molybdenum-containing periplasmic enzyme TorA as a terminal reductase. TMAO reductase (TorA) contains a bis-molybdopterin guanine nucleotide (bis-MGD) cofactor. TorD is a bis-MGD-binding chaperone that inserts the bis-MGD cofactor after its completion into apo-TorA. TorA belongs to the DMSO reductase family of molybdoenzymes and can reduce only TMAO, unlike *R. capsulatus* DorA, the DMSO reductase in this bacterium which reduces both *N*- and *S*-oxides [4].

TorC is a 43 kDa cytochrome that is largely hydrophilic but anchored to the membrane by a sequence of 20 hydrophobic amino acids at its N-terminus [5,6,7]. The protein contains five hemes that face the periplasm. Heme binding to the five consensus CXXCH motifs occurs in the periplasm by a mechanism involving the c-type cytochrome maturation machinery encoded by the *ccmABCDEFGH* genes [8]. TorC is required for anaerobic growth in the presence of TMAO; it shuttles electrons from the menaquinone pool to TorA in the periplasm [7]. TorC contains an N-terminal domain (TorC_N_) and a C-terminal domain (TorC_C_) of a similar size; TorC_N_ contains the membrane anchor followed by four heme-binding sites; meanwhile, TorC_C_ contains the fifth heme-binding site, and this domain has been proposed to donate electrons directly to TorA [7]. Interaction studies of TorC and TorA have shown that only TorC and TorC_N_ strongly bind TorA [7]. Apo-TorC binds to the periplasmic domain of TorS (TorS_N_), thereby preventing the phosphorylation of TorR [9].

The *c*-type cytochrome TorC is essential for the electron transfer from the menaquinone pool to TorA [7,10]. From the sequence homologies, it belongs to the NirT/NapC family of multiheme c-type cytochromes, which is composed of the N-terminal tetraheme globular domain, as found in the c-type cytochromes of nitrite and nitrate reduction systems [11,12]. The N-terminal segment of TorC is homologous to the proteins NirT and NapC, whereas the C-terminal segment is only present in DMSO/TMAO respiratory systems [6]. The *c*-type cytochromes within the NirT/NapC family—NapC, DorC, and TorC—have been previously characterized, and the redox potentials for all heme centers of NapC (tetraheme), DorC (pentaheme), and TorC have been determined [7,13]. The membrane-associated full-length TorC and its N- and C-terminal domains have been solubilized and purified from membrane fractions from *E. coli* for biochemical characterization [7,9].

In this work, we designed an expression system for the production of a stable soluble form of multiheme-containing TorC in *E. coli*. This stable and soluble TorC has its membrane anchor segment deleted but still contains both TorC domains to study their functionality. This provides, for the first time, the purification of a soluble pentahemic cytochrome-*c* from *E. coli*. A soluble TorC that can be easily purified will be useful for further biochemical and structural characterizations of TorC, which will require larger amounts of proteins. To validate the functionality of soluble TorC, we investigated its redox activity and interaction with TorA. We then established an activity assay that uses the physiological electron transfer pathway. The goal was to replace non-physiological electron donors such as methylviologen or benzylviologen, which are intrinsically instable and, therefore, might imply higher enzyme activities than are actually present.

Even though the role of TorA has been documented previously, it is still not known how electron transfer from the second domain of TorC (TorCc) to the active site of TorA occurs in detail. Our focus was to investigate the interaction between TorA and soluble TorC by structural analysis and chemical crosslinking to obtain insights into the electron transfer mechanism within the TMAO respiratory complex.

## 2. Results

### 2.1. Expression and Purification of Soluble Periplasmic TorC in E. coli HM125

**Figure 1:**
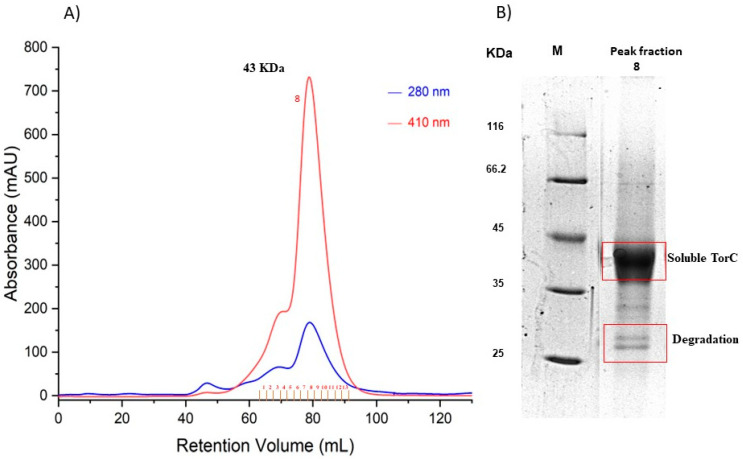
Purification of soluble TorC using gel filtration chromatography. (**A**) shows the gel filtration profile on a 16/600 Superdex 200 prep-grade column equilibrated in 50 mM Tris- HCl, pH 7.6 of the Ni-NTA-purified TorC (loaded approx. 20 mg) recorded at 280 nm (blue) and 410 nm (red). The peak eluting at 410 nm represents the absorbance of the heme-containing TorC. Fraction numbers are indicated at the respective volumes in red. (**B**) shows the 12% SDS-PAGE analysis of the main peak—No. 8—after gel filtration chromatography (elution volume 80 mL). M: molecular mass standard.

For the purification of soluble heme-containing TorC from the periplasm, we cloned the gene encoding TorC, lacking sequences encoding the N-terminal 20 amino acid membrane anchor into vector pBAD-pelBSS, which adds the periplasmic signal sequence from PelB to the N-terminus and a 6xHisTag to the C-terminus (pTorC-SS). Since *E. coli* can only synthesize limited amounts of *c*-type cytochromes, we co-transformed *E. coli* with both the plasmid pTorC-SS and the plasmid pEC86 containing the cytochrome-*c* maturation genes *(ccmABCDEFGH* gene cluster) to enhance heme *c* insertion in the periplasm [14]. The homologous expression of recombinant TorC was investigated under aerobic and anaerobic conditions. We obtained the highest expression levels when the cultures were grown aerobically in the presence of plasmid pEC86. Further, we tried different expression strains since TorC was degraded during purification. The proteolytic degradation was reduced to a minimum when both plasmids were introduced in the *E. coli* strain HM125, which lacks the periplasmic DegP protease [15]. The obtained TorC fraction after Ni-NTA affinity chromatography was further purified using gel filtration. The gel filtration chromatogram showed the elution of the heme-containing TorC, which was characterized by absorbance at 410 nm and whose purity was confirmed by SDS-PAGE. TorC was eluted mainly as a monomer from a Superdex 200 gel filtration column with a molecular mass of 43 kDa (Figure 1A). SDS-PAGE revealed a strong band for TorC at 43 kDa, and the smaller bands below that were degradation products of TorC as elucidated by mass spectrometry (Figure 1B). No other proteins were copurified with TorC, so we estimated a purity of 95%.

### 2.2. Characterization of Purified Soluble TorC

**Figure 2:**
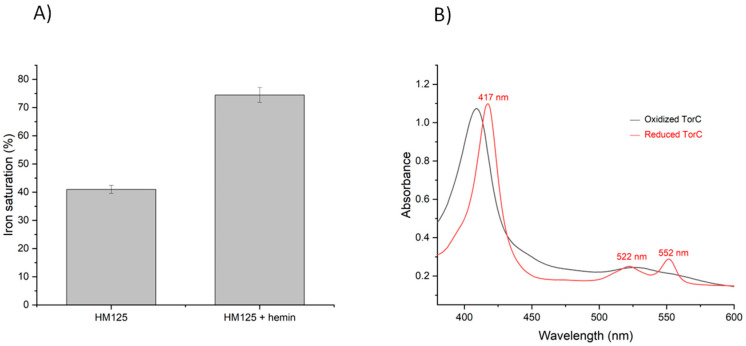
Characterization of purified soluble TorC. (**A**) ICP-OES analysis of purified soluble TorC showing the iron saturation of the protein, where 100% iron saturation corresponds to the presence of five hemes in the purified protein. (**B**) UV-Vis spectra of purified soluble TorC (10 µM) in the oxidized form (black line) and sodium dithionite (0.01 mM)-reduced form (red line) in 50 mM Tris-HCl pH 7.6.

The content of hemes within the purified protein was quantified by measuring the iron concentration using ICP-OES. ICP revealed that iron saturation was only about 42% when assuming five hemes in the protein. To improve heme saturation, we added hemin during expression, which increased the iron saturation to 75% (Figure 2A). Further, the UV–visible spectrum of purified TorC was measured in its oxidized and dithionite reduced forms. The spectrum shows the characteristic features of *c*-type cytochromes, with a major Soret band at 410 nm in the oxidized form, which, after dithionite reduction, shifted to 417 nm, accompanied by the appearance of the β-band at 522 nm and the α band at 552 nm (Figure 2B). Ascorbate reduced TorC to a lower extent than dithionite (Figure S1), which points to at least one heme with a more positive redox potential in soluble TorC.

### 2.3. Size Exclusion Chromatography of TorC and TorA

**Figure 3:**
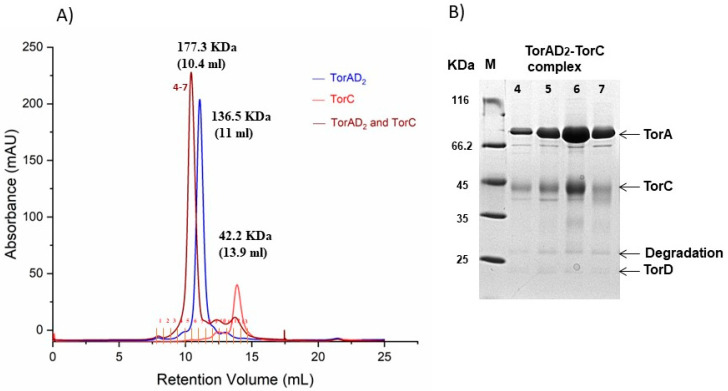
Analysis of TorAD_2_-TorC complex formation using gel filtration. (**A**) Overlay of the gel filtration profiles of TorAD_2_ (blue), soluble TorC (red), and a 1:1 TorC/TorAD_2_ (brown) mixture (30 µM, each mixed in 50 mM Tris- HCl, pH 7.6), resulting in the formation of a TorC/TorAD_2_ complex (brown); and (**B**) SDS PAGE analysis of fractions 4–7 from the major peak of the complex eluted at 10 mL. M: molecular mass standard.

TorC purified from membrane fractions was shown to interact with TorA [7]. Here, we employed analytical size exclusion chromatography to detect complex formation between soluble TorC and TorA. TorA was purified in a 1:2 stoichiometry with TorD (molecular mass of 22.57 kDa), forming a complex of 136.5 kDa to increase its stability, as reported previously [16]. As shown in Figure 3A, in the presence of excess soluble TorC, all TorAD_2_ was converted to a larger molecular mass complex of 177.3 kDa, which corresponded to a complex of TorC with TorAD_2_. The protein fractions were also analyzed by SDS-PAGE, and both TorA and TorC bands were detected in the peak corresponding to the TorC-TorAD_2_ complex in Figure 3B.

**Figure 4:**
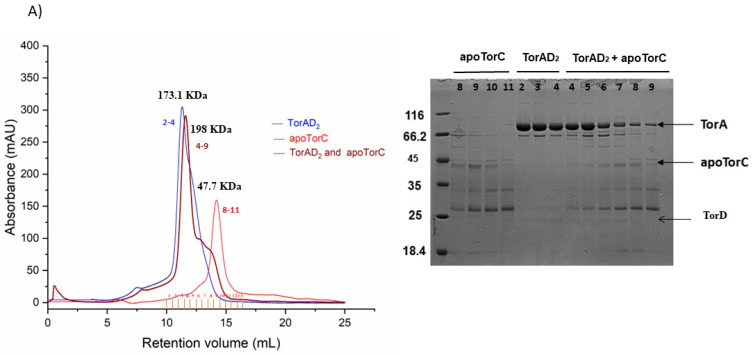

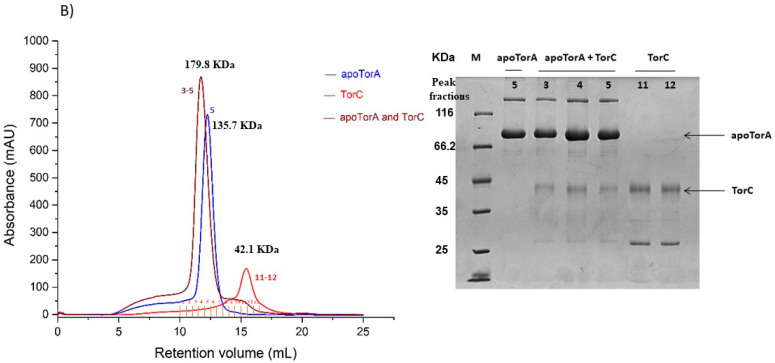
Analysis of TorAD_2_-apoTorC and apoTorA-TorC complex formation using gel filtration. Overlay of gel filtration profiles and SDS PAGE analysis of the fractions from the main complex peaks. The molecular masses of individual proteins and complexes formed were determined by an analytical Superdex 200 10/300 GL column. The concentrations used were 30 µM for each protein in 20 mM phosphate buffer, with pH 7.4. (**A**) apoTorC-TorAD2 and (**B**) apoTorA-TorC, together showing the complex elution peak (in brown).

Heme-containing holo-TorC was shown to directly interact with TorA for electron transfer [7]. To characterize the apo-form of soluble TorC, the binding between apo-TorC and TorAD_2_ was also analyzed by gel filtration. The chromatogram did not show a shift in the complex peak, and there was no big change in the apoTorC band in the main elution peak with TorAD_2_ on the SDS-gel (Figure 4A). In contrast, apo-TorA interacted well with holo-TorC (Figure 4B), as previously reported [9,17].

### 2.4. Analysis of Protein–Protein Interactions by SPR and Microscale Thermophoresis Measurements

To analyze the dissociation constants of interactions of TorC with TorA and apo-TorC with TorS_N_ (periplasmic detection domain of TorS), SPR measurements were employed.

TorAD_2_, apo-TorA, TorC, and apoTorC were immobilized on a CM5 chip via amine coupling. As binding partners (156 nM to 10 µM), we used purified soluble TorC and TorS_N_. The resulting sensograms reflect the association and dissociation of the two analyzed proteins. They confirm the interaction of soluble TorC with TorA and apo-TorA and the interaction of TorS_N_ with apo-TorC. The kinetic data were fitted in the plot based on the best 1:1 binding model. The mean *K*_D_ values obtained from three independent SPR measurements for the protein pairs are listed in Table 1 (SPR sensograms are shown in Figure S2). An interaction of apo-TorC with TorS_N_ was detected, showing that the purified apo-TorC was correctly folded. The interaction of holo-TorC with TorS_N_ was in the same range as the interaction with apo-TorC, implying that the interaction site with TorS was not influenced by the heme content of TorC. Conclusively, since apo-TorC did not interact with TorA, it was available to interact with TorS_N_ instead and, thereby, block the phosphorylation of TorR. For the interaction of TorAD_2_ with TorC or apo-TorA with TorC, similar *K*_D_ values were obtained, indicating the same interaction site and a similar folding of apo-TorA and holo-TorA.

As an additional method, we used microscale thermophoresis to confirm the *K*_D_ values obtained by SPR. In MST, one of the binding partners (TorAD_2_) was labeled with fluorescent dye (NT-647-NHS) that was kept at a constant concentration and titrated against increasing concentrations of soluble TorC as the ligand (from 0.065 μM to 208 μM). The resulting MST traces from the binding experiment showed no aggregation and adsorption of the protein within the capillary and confirmed the interaction of TorAD_2_ and soluble TorC (Figure S3). The sigmoidal dose curve obtained from the plot of normalized fluorescence and different ligand concentration was fitted based on a *K*_D_ model and yielded a *K*_D_ of about 10.7 × 10^−7^ ± 0.217 M, which was in the same range as the *K*_D_ obtained by SPR.

### 2.5. Kinetic Parameters of TMAO Reductase

Artificial electron donors such as dithionite have been observed to directly reduce the molybdenum in Moco [18] and possibly affect the activity of the enzyme. Moreover, artificial electron donors methylviologen and benzylviologen are intrinsically unstable. For these reasons, we wanted to establish an assay without such artificial electron donors using, instead, the physiological electron transfer pathway consisting of menadiol and soluble TorC. Here, TorA activity could directly be detected by the quantification of produced TMA. TorC was proposed to act as a mediator between the menaquinone pool and the terminal reductase TorA. The electrons were transferred from menaquinones to the active site of TorA, where TMAO reduction occurred. Commercially available menadione is an analog for menaquinone with the same hydrocarbon ring backbone but lacking the side chain. The reduced form of menadione (menadiol) was obtained by treating it with sodium borohydride in an anaerobic chamber (Figure S4).

To detect the electron transfer from menadiol via TorC to TorA, we established an assay to detect the TMA produced from TMAO reduction by TorA. The detection and quantification of TMA were performed by GC-MS [19]. The produced TMA in the assay was derivatized to form N,N-dimethyl-2,2,2-trichloroethyl carbamate, which could be separated by gas chromatography, and the fragmented ions produced were detected by the attached mass spectrometer (Figure S5A). The fragment ions were quantified using selected ion monitoring (SIM method). Fragment ions at *m*/*z* 72 formed from derivatized TMA were observed, and the retention time of the peak was about 8.7 min (Figure S5B). The similarity search result in the GC-MS post-run analysis software (Version 4.41) confirmed that this fragment ion corresponded to the derivatized form of TMA.

The assay for measuring TMAO reductase activity was carried out under anaerobic conditions using menadiol as an electron donor. The UV–visible spectrum of TorC reduced by menadiol was similar to that obtained after reduction with dithionite. Moreover, the addition of TMAO to the assay did not modify the spectrum, while the addition of TMAO and TorA resulted in the oxidation of soluble TorC. This result shows that menadiol can reduce soluble TorC, which can be re-oxidized by TorA in the presence of TMAO (Figure S6). Further, different TMAO concentrations were used in the range of 5–250 µM to determine the kinetic parameters of the enzyme. Using a calibration series of TMA standards with given peak areas, the produced TMA was quantified by linear regression analysis. For comparison, dithionite-reduced benzyl viologen and methyl viologen as direct electron donors for TorA were additionally used to determine and compare the kinetic constants of the enzyme. The results are shown in Table 2, and the hyperbolic saturation curves and Lineweaver–Burk linearizations are shown in Figure S7. The results indicate clearly that a reduction of TMAO by TorA occurs via soluble TorC, using menadiol as the electron donor. The *K*_M_ values determined for TMAO with reduced menadiol (9.8 µM), benzyl viologen (22 µM), and methyl viologen (32 µM) as electron donors did not vary substantially. However, the calculated *k*_cat_ values were much lower with menadiol as the electron donor. Differences in the *k*_cat_ values between benzyl viologen and methyl viologen were likely due to the lower reduction potential of menadiol (E°^’^ menadiol/menaquinone = −189 mV) compared to methyl viologen (E°^’^ MV^2+^/MV^+^ = −450 mV) and benzyl viologen (E°^’^ BV^2+^/BV^+^ = −374 mV). The higher reduction potentials of the latter allowed for a faster and more direct electron transfer to bis-MGD.

### 2.6. Chemical Crosslinking of TorA and TorC

We performed the chemical crosslinking of TorA and TorC, followed by mass spectrometry, to detect the interaction site that promoted electron transfer between both proteins. We used DSPP as the enrichable crosslinker that crosslinked exposed lysine residues at a distance of 4.8 Å. The same experiments were also performed with TorC or TorA alone. The identified intra- and intermolecular crosslinking sites are depicted in Table 3.

### 2.7. Crystal Structure of TorA and AI-Based Structural Model for the TorA-TorC Complex

**Figure 5:**
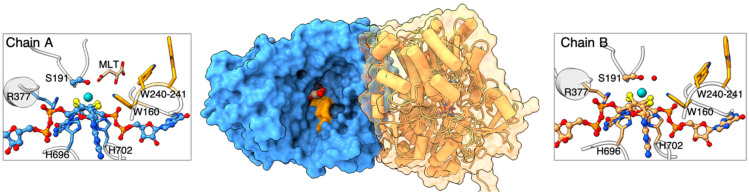
Crystal structure of TorA and active site composition. The center panel shows the two molecules in the asymmetric unit with chain A shown as a blue surface and chain B as a transparent gold surface, with a cartoon representation underneath. Chain A has a solvent-accessible active site and binds a malate molecule (shown as spheres with a cream color for carbon and red for oxygen) from the crystallization solution. The orange surface indicates the positions of three tryptophan residues (W160, W240, and W241) lining the active site pocket. The left and right panels show the active sites for chains A and B, respectively, with the bis-MGD cofactor and coordinating serine 191 (S191) depicted as a ball-and-stick structure colored light blue (**left panel**) or gold (**right panel**) for carbon, red for oxygen, dark blue for nitrogen, orange for phosphor, and yellow for sulfur. Neighboring residues to the molybdenum anions (depicted as cyan spheres) are shown as sticks. The malate (MLT) bound to the active site in chain A (**left panel**) is shown as a stick with carbon in a cream color and oxygen in red. The distance between the carboxylate group and Mo is 3.5–3.9 Å. In chain B (**right panel**), the entrance to the active site is blocked by chain B, and we find a water molecule (red sphere) at 2.6 Å from Mo.

Having established the conditions for the formation of a stable TorC-TorA complex, we set up crystallization trials with TorA-TorC and TorA alone to analyze the molecular basis for electron transfer. Although we were unable to obtain diffracting crystals for TorA-TorC, we were able to solve the crystal structure of TorA by molecular replacement with dimethyl sulfoxide reductase (DMSO reductase) from *Rhodobacter capsulatus* (PDB 1dms; ref. [20]) as a homologous search model. The two chains present in the asymmetric unit were oriented toward each other such that one molecule (chain A) covered the entrance to the other molecule’s (chain B) active site (Figure 5). Both active sites had clear electron density for the Mo ion coordinated by bis-MGD and serine 191 (1.7 Å from Mo), but no obvious density for a sulfido ligand [21]. The sulfido-containing bis-MGD is highly sensitive to oxygen [21] and may have been damaged during the long time required for crystallization (>1 month). While the solvent-accessible active site (chain A) had a malate moiety at 2.8–2.9 Å from Ser191(OH) and at 3.5–3.9 Å from Mo (distance from carboxyl group to Ser and Mo), the covered active site (chain B) had a water molecule at 2.6 Å from Mo and at 2.2 Å from Ser191(OH). We could not discard a partially occupied sulfido ligand at this position.

**Figure 6:**
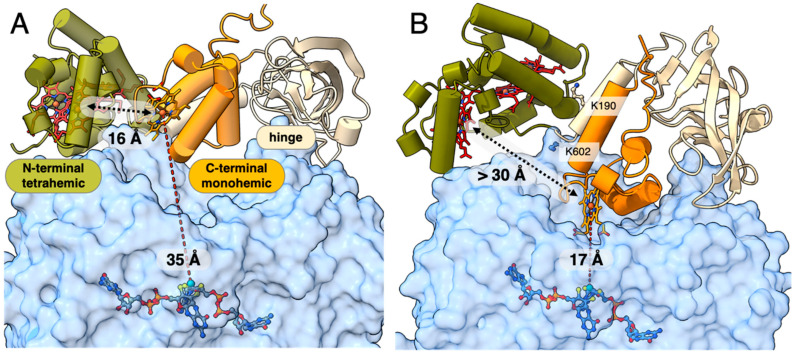
Models for electron transfer within the TorA-TorC complex. (**A**) Model generated by AlphaFold-multimer. TorC is shown as a cartoon with TorC_N_ colored in olive, the hinge domain in cream, and TorC_C_ in orange. The crystal structure model of TorA is shown as a transparent blue surface with the bis-MGD cofactor depicted as a ball-and-stick figure with the same color scheme as in Figure 5. Hemes are added to TorC by superposition with the TorC model generated by AlphaFold3 and are shown as ball-and-stick figures with the Fe ions colored in orange. The distance between TorC_C_-Heme (Fe ion) and Mo is 35 Å (dashed red line), and the distance to the nearest heme in TorC_N_ is 16 Å (dashed black line). (**B**) Model generated by AlphaLink2. The predicted template modeling (pTM) score and the interface predicted template modeling (ipTM) score are, respectively, 0.85 and 0.83. The side chains from the validated crosslink between TorA:K602 and TorC:K190 are shown as ball-and-stick figures. The orientation of the complex is slightly different from panel A for better visualization of the shorter distance between TorC_C_-Heme (Fe ion) and Mo in TorA (17 Å, dashed red line). The distance between TorC_C_-Heme (Fe ion) and the nearest heme in TorC_N_ is now >30 Å (dashed black line).

In the absence of a TorA-TorC structure, we used the AlphaFold-multimer [22,23] to generate 10 models of the complex. All 10 models showed almost identical TorC and TorA placements but a higher per-residue local confidence for TorA (Figure S8A). The first 50 and last 10 residues of TorA were modeled with low confidence values, indicating high flexibility. This was supported by the crystal structure in which these residues were not defined in the electron density. We therefore replaced the generated TorA model by the TorA crystal structure for further analysis of the TorA-TorC complex. Superposition with NirFHA (PDB 2vr0, [24]) placed the five hemes in the correct CXXCH binding motifs. TorC comprised an N-terminal domain (1–143) binding four hemes (TorC_N_), a hinge or linker domain (144–274), and a C-terminal domain (275–351) binding one heme. From a previous work [7], we knew that the N-terminal tetrahemic domain (TorC_N_) was postulated to bind to TorA and relay the electrons from the menaquinone pool to the C-terminal monohemic domain (TorC_C_), which was postulated to transfer the electrons to the active site in TorA. The TorA-TorC complex model positioned TorC_C_ above the substrate-binding funnel but approximately 35 Å away from the Mo (Figure 6A). To rule out the possibility that the absence of hemes could affect the calculation of the complex, resulting in this long-range interaction, we used the recently released AlphaFold3, which allowed for the prediction of both proteins and ligands, but all five models generated had practically identical TorA-TorC interactions. In parallel, we included the TorA-TorC crosslinks in AlphaLink2 [25] (Figure 6B). Compared to the AlphaFold-multimer model (Figure 6A), the resulting model showed TorC rotated by about 20 Å along the front of TorA, positioning TorC_C_ in the substrate-binding funnel at a reasonable distance for electron transfer to Mo (17 Å, Figure 6B). TorC_N_ also rotated 12 Å away from TorC_C_. To gain further insight into the dynamics of the TorA-TorC complex, we generated 25 new TorA-TorC models using the AlphaFold2-multimer. Fourteen models were similar to the previously generated models (example in Figure 6A) and one model was practically identical to the complex generated by AlphaLink2 (Figure 6B). The remaining ten models exhibited TorC_C_ and hinge domains arranged in a manner analogous to the complex generated by AlphaLink2 (Figure 6B) and yet with a gradient of varying rotations of TorC_N_ toward TorC_C_. This provided us with snapshots of the interdomain dynamics during the electron relay with TorC_C_ shifting toward TorC_N_ to become reduced and subsequently moving into the active site cavity of TorA for electron transfer. The interdomain dynamics of TorCc were most likely supported by the hinge domain of TorC.

Superposing both states, the one with TorC_C_ at 35 Å from the Mo ion and the one with TorC_C_ at 17 Å from the Mo ion, which we called *TorC_C_ out* and *TorC_C_ in*, we could envisage a path for the electron relay: when the complex was in the *out*-conformation state, TorC_C_-Heme was 16 Å from the nearest heme in TorC_N_ (Figure 6A), allowing the transfer of electrons from TorC_N_ to TorC_C_. In the *in*-conformation state, this distance increased to >30 Å as TorC_N_ rotated away and TorC_C_ moved down the substrate-binding funnel, positioning its heme at a distance of 17 Å for electron transfer to Mo (Figure 6B).

## 3. Discussion

We succeeded in purifying soluble TorC from *E. coli* in an active form. We were able to obtain soluble TorC by cutting off the N-terminal membrane anchor and adding the periplasmic PelB signal sequence instead, so that soluble TorC could mature in the periplasm. To facilitate purification, we added a C-terminal His_6_-sequence. The heme-loading of soluble TorC was increased by the co-expression of the *ccm*-operon and, in addition, by adding hemin during expression. With this, we were able to obtain soluble TorC with an almost 75% heme saturation. The stability of TorC was increased during purification using strain HM125, which lacked the periplasmic protease DegP. Soluble TorC could be purified as a monomer with a purity of almost 95%. Soluble TorC was used to further characterize and understand the electron transfer pathway to TorA. Previously, by using TorC-purified domains, it was shown that only TorC_N_ bound efficiently to TorA (*K*_D_ of 4.5 × 10^−8^ M), while TorC_C_, which fed TorA with electrons, showed no significant binding to it. This led to a proposed model in which the binding of TorC_N_ to TorA allowed for the correct positioning of TorC_C_ to transfer electrons to the catalytic site of TorA. The results of soluble TorC mainly confirmed the previously obtained results and showed that TorD copurified with TorA did not interfere with the binding of TorC. However, when TorC was lacking hemes, it was in a conformation unable to bind to TorA, so it was available for binding to TorS instead, then blocking the phosphorylation of TorR. Further, we could confirm that soluble apo-TorC bound itself to TorS_N_, and we obtained the same binding constant of TorC to apo-TorA as to holo-TorA, indicating the same folds in both proteins. Altogether, these results indicate that soluble TorC behaves similarly to the native membranous protein, and, thus, through its larger production, it could be used for further characterizing the electron transfer during TMAO respiration. Our AI-based TorA-TorC complexes, combined with the crosslinks and crystal structure of TorA, support an interdomain dynamic in which TorC_C_ pivots between TorC_N_ and the bottom of the substrate-binding funnel of TorA during the electron relay.

One of this study’s purposes was to establish an enzyme assay illustrating the physiological electron transfer pathway with menadiol of TorC to TorA for the reduction of TMAO. This assay was established to replace the commonly used assays employing benzyl viologen or methyl viologen. As both mediators are intrinsically unstable, inaccurate results are often obtained. Further, in previous studies, dithionite used as a reductant has been observed to reduce the bis-MGD of DMSO reductase DmsABC and TorA directly, but binding or interference with the bis-MGD cofactor has also been suggested, hence interfering with the activity and substrate affinity of the enzyme [18]. In our study, we established an assay using soluble TorC and menadiol as more physiological reductants of TorA. The reduced form of menadione (menadiol) was obtained by treating it with sodium borohydride. TorA activity was monitored by measuring the product formation using GC-MS. After the addition of TorA and TMAO to reduced TorC by reduced menadiol, TorC was re-oxidized (Supplemental Figure S6). For comparison, the commonly used artificial electron donor dithionite that reduced benzyl viologen or methyl viologen was also used to reduce TorA. In this assay, the re-oxidation of benzyl viologen and methyl viologen was monitored. While the *K*_M_ values obtained with reduced menadiol and viologen compounds as electron donors were in the same range, the calculated *k*_cat_ values were much lower when using the physiological electron donor. It seems that the reduction of TMAO by menadiol via reduced TorC was slower compared to the direct reduction of TorA by dithionite-reduced benzyl viologen. Likely, when using benzyl viologen or methyl viologen, the decay of both mediators and their reaction with oxygen are monitored alongside the specific enzymatic reaction. Therefore, the kinetic constants of the assays when benzyl viologen and methyl viologen are used must be considered carefully. Another explanation is the different redox potentials of the three electron donors. Finally, one could also argue that benzyl or methyl viologen can bind in a closer vicinity to the bis-MGD cofactor, since the mediators are smaller, while the binding site of TorC is further away. A model was proposed in which the TorC_C_ domain was located at the substrate-binding funnel, thereby hindering the access of the substrate to the active site. However, we could not compare the *K*_M_ and *k*_cat_ values for membrane-bound TorC, since they had not been reported yet. It is possible for membrane-bound TorC to have lower k_cat_ values based on a reduced flexibility of the protein when bound to the membrane. Overall, the previously proposed model regarding the flexibility and movement of TorC toward the active site of TorA for electron transfer [7] was confirmed in our study. This electron transfer between a heme cofactor and bis-MGD is surprisingly similar to the electron transfer pathway between the heme-domain and Moco in sulfite oxidase [26] which also requires a domain movement of the heme-domain (cytochrome b5) to draw closer to the Moco in this enzyme. This might be a common way for hemes to interact with other cofactors to ensure a direct and fast electron transfer.

## 4. Materials and Methods

### 4.1. Bacterial Strains, Plasmids, Media, and Growth Conditions

The strains and plasmids used in this study are listed in Table 4.

*E. coli* cultures were grown in LB medium. When required, ampicillin (150 µg/mL), kanamycin (50 µg/mL), or chloramphenicol (50 µg/mL) was added to the medium during growth. Protein expression from the *ccmABCDEFGH* operon cloned into the pACYC184 vector was induced by the addition of 0.005% L-arabinose. For the expression of soluble TorC, the truncated gene (deletion of the first 60 nucleotides) was cloned into vector pBAD-pelBSS, adding the corresponding signal sequence of the *pelB* gene to the 5′end and a sequence encoding a 6 × HisTag to the 3′end using appropriate primers. Expression was induced by the addition of 0.005% arabinose for 16–18 h. The growth medium was also supplemented with 10 μM hemin.

For the production of apoTorC, the “soluble *torC*” gene was expressed without the *ccm* gene cluster from pEC86. In addition, the final concentration of arabinose to induce expression was increased to 0.02% and expressed for 7–8 h without any additional hemin.

### 4.2. Purification of Soluble TorC

The cells were harvested and then resuspended in the washing buffer (10mM imidazole, 300 mM NaCl, 50 mM Na_2_H_2_PO_4_, and pH 8.0) with the addition of complete EDTA-free protease inhibitors (Roche, Germany). Cell lysis was performed on a cell disruptor system followed by centrifugation at 4 °C at 13,500 rpm. Next, the cleared lysate was applied onto a Ni-NTA (Ni-nitrilotriacetate) column (0.4–0.5 mL per liter of cell culture) and subsequently washed with 15 column volumes of 10 mM imidazole and 20 mM imidazole washing buffer. The bound TorC was then eluted with the buffer containing 250 mM imidazole (250 mM imidazole, 300 mM NaCl, 50 mM NaH_2_PO_4_, and pH 8.0). The buffer was changed to 50 mM Tris-HCl with a pH of 7.6 using PD10 columns, and the protein was concentrated using Microcon concentrators. The Ni-NTA-purified TorC was further purified by size exclusion chromatography on a 16/600 Superdex 200 prep-grade column (GE Healthcare, Little Chalfont, UK). The fractions containing TorC were collected, concentrated, and stored at −80 °C.

### 4.3. Protein Purification of TorA

TorA and apo-TorA were purified following published procedures [31].

### 4.4. Protein Purification of TorS

TorS_N_ was purified following published procedures [7].

### 4.5. Metal Analysis

Metal analysis was performed using a Perkin-Elmer Optima 2100DV inductively coupled plasma-optical emission spectrometer (Perkin-Elmer, Fremont, CA, USA). The iron and molybdenum contents of the TorC and TorA proteins, respectively, were quantified using 10 µM of the protein in a volume of 500 µL in Tris-HCl mixed with 500 µL 65% nitric acid in falcon tubes wet-ashed overnight at 100 °C. Afterwards, 4 mL of water was added to each tube. As a reference, the multi-element standard XVI (Merck) was used.

### 4.6. TorA Activity Assays

The activity of TorA was measured under anaerobic conditions by monitoring the oxidation of pre-reduced benzyl viologen or methyl viologen at 600 nm. The assay was conducted in 100 mM Sörensen phosphate buffer with a pH of 6.5, containing 2.5–250 µM TMAO and 0.4 mM BV or 0.4 mM MV, which was reduced by the addition of sodium dithionite. Measurements began after the addition of 3 nM of TorA to the reaction.

For the menadiol-based assay, the activity of TorA was calculated by measuring TMA formation with GC-MS. The assay was conducted anaerobically in 100 mM Sörensen phosphate buffer at a pH of 6.5, which contained 5 µM menadiol (pre-reduced with 2× sodium borohydride), 3 µM soluble TorC, and 7.5 mM of TMAO, and the reaction was catalyzed by the addition of 3 µM TorA. Following the reaction, the samples were treated with acetonitrile to precipitate the proteins and then centrifuged at 13,000 rpm for 15 min. The supernatant was collected and processed further for TMA derivatization.

#### Detection of TMA Using GC-MS

The detection and quantification of derivatized TMA was performed using the GC-MS QP2010SE instrument (Shimadzu Europa; Duisburg, Germany) with a DB-WAX UI column (30 m × 0.32 mm × 0.25 µm, Agilent, Santa Clara, CA, USA). The conditions for the GC program were as follows: temperature gradient from 50 °C to 220 °C, increasing at a rate of 30 °C per minute; split injection mode; He, carrier gas; and column flow of 1.65 mL/min. The fragments formed by electron ionization were quantitated using selected ion monitoring (SIM method) adapted from ref. [19]. The fragmented molecular ions at *m*/*z* 72 (from derivatized TMA) and *m*/*z* 76 (from internal standard TMA-d_9_) were observed. The derivatized product from TMA and N,N-dimethyl-2,2,2-trichloroethyl carbamate had a retention time of 8.7 min.

### 4.7. Reduction of the Menaquinone Analog

Menadione was purchased from Sigma Aldrich and was reduced with sodium borohydride in an anaerobic chamber. A stock solution of 50 mM menadione was prepared in ethanol. For the reduction of menadione, 1 mM menadione was prepared in 20 mM phosphate buffer with a pH of 7.4, and 0.1 mM sodium borohydride was added stepwise until a full reduction occurred, which was measured in the spectrophotometer within the range of 200 nm to 400 nm. The reaction mix was incubated for about 10 min, and then the excess borohydride was removed via the addition of HCl.

### 4.8. Interaction Analysis with MST

The Nanotemper instrument (Monolith NT.115) was used to analyze the interaction of TorA and TorC using microscale thermophoresis. One of the interaction partners of TorA was labeled with NT-647-NHS fluorescent dye, (Nanotemper, Munich, Germany) which reacts with the primary amines in proteins. Serial dilutions for ligandN TorC (stock concentration 65 µM) were prepared in 16 PCR tubes, and all were titrated against the fixed concentration of TorA (5 nM). Measurements were performed at 25 °C in premium capillaries with 5% excitation power and 40% MST power. The MST analysis software (MO.Affinity Analysis 3 software) was used to evaluate the binding isotherm curve and the *K*_D_ value for the interaction.

### 4.9. Surface Plasmon Resonance (SPR) Measurements

All binding experiments were performed on an SPR-based BiacoreTM T200 instrument employing CM5 sensor chips at a temperature of 25 °C and a flow rate of 30 µL/min using the Biacore control T200 software and the evaluation T200 software (GE Healthcare). The autosampler rack containing the samples was cooled throughout the measurements to 8 °C. Proteins were immobilized with the following recovery units: BSA, 500–1000; TorC, 1581; TorA, 150–700; apo-TorC, 551; and apo-TorA, 400–1100. The proteins for each immobilization process were the result of independent purifications. Regarding the running buffer, 20 mM phosphate, 150 mM NaCl, and 0.005% (*v*/*v*) Tween 20, with a pH of 7.4, were used. Analytes with concentrations of 156 nM–20 µM were injected for 4.5 min at a flow rate of 30 µL/min followed by 15 min dissociation and regeneration of the sensor surface with 50 mM HCl for 1 min. Bovine serum albumin (BSA) served as the control ligand. Binding curves were corrected by subtracting buffer injection curves for both flow cells.

### 4.10. Size Exclusion Chromatography of the TorC-TorA Complex

Size exclusion chromatography was performed at 8 °C using a Superdex 200 10/300 GL (GE Healthcare) equilibrated in 50 mM Tris-HCl (pH 7.6) on an Åkta purifier system (GE Healthcare). The standard protein marker (Biorad) was used to determine the molecular weight of the complex. Mixtures containing 3 µM TorC and 30 µM TorA were incubated for 30 min at 25 °C prior to gel filtration.

### 4.11. Derivatization and Detection of Trimethylamine (TMA)

The derivatizing agent 2,2,2-trichloroethyl chloroformate for TMA was purchased from Sigma Aldrich. TMA was derivatized to form the compound N,N-dimethyl-2,2,2-trichloroethyl carbamate, which was then isolated in gas chromatography, followed by electron ionization with MS. For derivatization, toluene (250 µL) was added to the samples followed by KOH (0.5 mL; 65% in water). The samples were incubated at 90 °C for 1 h, shaken vigorously, and then centrifuged at 13,000× *g* for 5 min at 4 °C. An aliquot (150 µL) of the cooled toluene layer was added to a vial containing 10 µL of 2,2,2-trichloroethyl chloroformate. The samples were incubated at 100 °C for 30 min. Methanolic alkali (250 µL) was then added after cooling down the samples, and the reaction mixture was mixed. Water (0.5 mL) was then added, and the samples were mixed and centrifuged at 13,000× *g* at 4 °C. Aliquots of about 50 µL were collected and diluted in a 1:3 ratio in HPLC vials and subjected to GC-MS.

### 4.12. Protein Crystallization and Structure Determination of TorA by Molecular Replacement

Crystallization trials for TorC-TorA (10–22 mg/mL in 50 mM Tris/HCl pH 7.6) and TorA (19–25 mg/mL in 150 mM Tris/HCl pH 7.2, 150 mM NaCl) were conducted using the sitting-drop vapor diffusion method at 18–20 °C under strictly anoxic conditions in an anaerobic chamber (Coy Laboratory Products, Grass Lake, MI, USA) with a gas atmosphere of 95% N_2_ and 5% H_2_. Initial commercially available screens were set using the nanodrop dispenser OryxNano (Douglas Instruments, Berkshire, UK) in 96-well MRC plates (JenaBiosciences, Jena, Germany) with varying drop volumes (1–1.4 µL) and a constant reservoir volume of 80 µL. Trials for TorC-TorA were not successful but TorA crystallized under different conditions. The best X-ray-diffracting crystals of TorA appeared after 1 month in 150 mM di-Na DL-malate at a pH of 5, 4 mM NaF, and 60% PEG 1000 (condition H3 in Pi-minimal screen, JenaBiosciences, Germany) and were directly frozen in liquid nitrogen. Diffraction data to 1.86 Å were collected at 100 K on beamline BL 14.1 (wavelength 0.9814 Å) operated by the Helmholtz-Zentrum at the BESSY II electron storage ring (Berlin, Germany [32]) and processed with XDSAPP [33]. The crystals belonged to space group P2(1), with two molecules per asymmetric unit corresponding to 56% solvent content. The initial phases were obtained from Patterson search techniques with Phaser in Phenix [34], using the structure from dimethylsulfoxide reductase (DMSO reductase) from *Rhodobacter capsulatus* (PDB 1dms; [20] as the search model (poly-Ala without cofactor). We could not model both chains’ first 48 residues and last 5 to 10 residues. The two chains were oriented toward each other so that one molecule (chain A) covered the entrance to the other molecule’s (chain B) active site. The solvent-accessible active site in chain A showed additional electron density near Ser191, which we modeled as a malate molecule, a component of the crystallization condition. Iterative cycles of manual model building and refinement were carried out respectively in Coot [35] and with phenix.refine in Phenix [34]. The final refinement run was performed with PDB-REDO [36]. Table S1 shows the statistics for crystallographic data and protein model refinement. Coordinates and structure factor amplitudes for TorA are available at the Protein Data Bank (PDB), www.wwpdb.org, under accession number 9H4T (DOI: 10.2210/pdb9h4t/pdb).

### 4.13. TorA-TorC Crosslinking

The buffer for purified TorA and TorC proteins was exchanged for HEPES buffer (30 mM HEPES, pH 7.4, 50 mM NaCl) using NAP5 columns (GE HealthCare). Crosslinking was performed in a volume of 50 µL (5 µM TorA, 5 µM TorC, 100 mM NaCl, 20 mM HEPES at a pH of 7.4, 1 mM MgCl_2_, 1 mM ascorbate, 0.05% TX-100, and 1 mM DSPP crosslinker (ThermoScientific, Waltham, MA, USA) for 1 h at RT, and the reaction was quenched by adding Tris-HCl buffer (pH 8) to a final concentration of 20 mM.

Samples were digested tryptically using the SP3 protocol [37]. An aliquot of the tryptic digest was desalted on STAGE-Tips [38] and directly analyzed by LC-MS for protein identification and x-link search database generation. The remaining sample was desalted on StageTips and resuspended in 150 µL buffer A (80% acetonitrile, 0.1% TFA). Phospho-enrichment using magnetic Fe-NTA beads (ThermoFisher) was performed as follows: On a magnetic rack, beads from 20 µL bead slurry were washed two times with buffer B (99.9% acetonitrile, 0.1% TFA) and two times using buffer A. The beads and the peptide solution were combined and incubated on a shaker for 20 min at RT. The supernatant was discarded, and the beads were washed three times using buffer A and transferred to a new tube. Bound peptides were eluted by incubation for 1 min, first with 30 µL E1 (5% NH_4_OH) and then with 30 µL E2 (5% NH_4_OH, 20% acetonitrile). The eluates were combined and directly transferred to 60 µL 10% TFA. Samples were then dried in a speedvac and cleaned up using StageTips before MS analysis.

MS analysis was performed on an LC-IM-MS (nanoElute2 coupled to a timsTOF Pro2, Bruker, Bremen) as described [39], using home-made columns (75 µm × 35 cm, Dr. Maisch C18aq, 1.9 µm material). The gradient for peptide elution ramped to a 30% organic phase within 34 min and then to 50% within 2 min, followed by washing steps. The samples were measured twice in the DDA-PASEF mode, with slightly different settings: firstly with standard settings as described, and secondly with broader 1/K_0_ (0.6–1.63 Vs/cm^2^) and stepping collision energy enabled (20–60 eV and 28–97 eV).

Data files for database generation were searched with MaxQuant v2.6.4.0 [40] with standard settings against the UniProt *E. coli* K12 proteome, including the modified TorA and TorC sequences. Identified proteins were ranked by iBAQ, and a FASTA file was generated using the 16 highest-ranking proteins.

Phospho-enriched sample files were searched against the generated database using MaxQuant [41] with the vendor raw data using DSPP and the TRIS-quenched modification as a non-cleavable crosslinker; FDR was controlled at 1% on all levels.

### 4.14. AI-Based Structural Model Calculations

Calculations with AlphaFold2 were performed with a locally installed version of AlphaFold 2.3.1 [23] with the AlphaFold-multimer model using GPU acceleration on an NVIDIA RTX A6000, with CUDA version 11.6. The used parameter templates were from 1 November 2021, and the models were minimized using the Amber99s force field. The heme groups were modeled based on superposition with NirFHA (PDB 2vr0). Calculations with AlphaFold3 (using heme c as the ligand) were performed via the AlphaFold Server (https://alphafoldserver.com) [42], and calculations with AlphaLink2 (integrate crosslinking MS results) were carried out through the Colab Notebook [25].

### 4.15. Structure Visualization

The graphics for the crystal structure of TorA and AI models for the TorC-TorA complex were visualized using ChimeraX [43].

## Figures and Tables

**Table 1:** SPR analysis using the Biacore T200 system. The equilibrium dissociation constants (K_D_) were determined using the BIAcore analysis software.

Immobilized Protein ^a^	Protein Partner ^b^	*K*_D_ ^c^ (M)
TorAD_2_	TorC	3.09 × 10^−7^
apo-TorA	TorC	2.66 × 10^−7^
holo-TorC	TorS_N_	1.03 × 10^−6^
apo-TorC	TorS_N_	9.43 × 10^−7^

^a^ Proteins were immobilized via amine coupling. ^b^ Proteins were injected using the KINJECT protocol, injecting samples in a concentration range of 0.4 to 20 µM. Cells were regenerated by the injection of 20 mMHCl. ^c^ *K*_D_ values were obtained by global fitting procedures for 1:1 binding.

**Table 2:** Kinetic constants for TMAO reductase using different electron donors.

Electron Donor	Menadiol ^a^	Benzyl Viologen ^b^	Methyl Viologen ^c^
*k*_cat_ (s^−1^)	0.145 ± 0.05	227.65 ± 46.3	464 ± 57.98
*K*_M_ TMAO (µM)	9.8 ± 2.53	22.06 ± 0.75	32.025 ± 4.56

^a^ Amount of TMA after derivatization, determined in 100 mM Sörensen buffer, pH 6.5, at 37 °C using 0.5–200 µM TMAO, and 5 µM reduced menadiol (32). ^b^ Oxidation of benzyl viologen at 600 nm (32), determined in 100 mM Sörensen buffer, pH 6.5, at 37 °C using 2.5–250 µM TMAO, and 400 µM benzyl viologen. ^c^ Oxidation of methyl viologen at 600 nm (32), determined in 100 mM Sörensen buffer, pH 6.5, at 37 °C using 2.5–250 µM TMAO, and 400 µM methyl viologen.

**Table 3:** TorA and TorC crosslinking sites using DSPP in four replicates.

Site TorAxTorC	Peptide Sequence 1	Peptide Sequence 2	PEP *	Occurrence in *n* = 4 Replicates	Charge
K602-K190	IWQEGVQQGKGR	KQFDELR	6.74 × 10^−6^	4	3, 4
K67-K190	ATVKDGR	KQFDELR	2.79 × 10^−5^	4	3
K67-K282	ATVKDGR	GDVQQQVKTLEK	5.78 × 10^−3^	2	3
K602-K190	RIWQEGVQQGKGR	KQFDELR	1.62 × 10^−2^	1	4
K67-K266	ATVKDGR	VLTQFPGKR	2.71 × 10^−2^	1	3
K551-K112	GIIAMK	FEAKR	3.47 × 10^−2^	1	2
**Site TorAxTorA**	**Peptide sequence 1**	**Peptide sequence 2**	PEP *	**Occurrence in *n* = 4 replicates**	**Charge**
K75-K67	FVAAKPFELDKYPSK	ATVKDGR	1.77 × 10^−5^	4	3, 4
K602-K67	IWQEGVQQGKGR	ATVKDGR	1.25 × 10^−4^	4	3
K81-K75	PFELDKYPSK	FVAAK	1.85 × 10^−4^	4	3, 4
K75-K67	FVAAKPFELDK	ATVKDGR	1.13 × 10^−3^	4	3
K81-K75	PFELDK	FVAAK	1.13 × 10^−3^	4	2
K473-K67	SVKLPPLK	ATVKDGR	1.84 × 10^−2^	2	3
**Site TorCxTorC**	**Peptide sequence 1**	**Peptide sequence 2**	PEP *	**Occurrence in *n* = 4 replicates**	**Charge**
K215-K300	GDKEASGSLLPASEVK	LQATAWMKK	1.83 × 10^−11^	4	3, 4
K184-K112	KQFDELR	FEAKR	4.42 × 10^−6^	4	2, 3
K260-K184	VLTQFPGKR	KQFDELR	2.32 × 10^−3^	4	3, 4
K276-K184	GDVQQQVKTLEK	KQFDELR	1.78 × 10^−3^	2	3
K155-K184	QMKVAAK	KQFDELR	2.35 × 10^−3^	4	3
K215-K184	GDKEASGSLLPASEVK	KQFDELR	3.46 × 10^−2^	1	3
K260-K155	VLTQFPGKR	QMKVAAK	3.65 × 10^−2^	2	3
K260-K112	VLTQFPGKR	FEAKR	3.80 × 10^−2^	1	3

* PEP: posterior error probability of the best-matching identification.

**Table 4:** Strains and plasmids used in this study.

*E. coli* Strains	Genotype/Characteristics	References
HM125	*KS272 degP eda rpoH/5*	[15]
BW25113(DE3)	*lacIq rrnBT14 ΔlacZWJ16 hsdR514* and *ΔaraBADAH33 ΔrhaBADLD78*	[27]
BL21(DE3)	*F-*, *ompT*, *rB-mB-hsdS*, *gal* (*λcIts857*, *ind1*, *San7*, *nin5*, and *lac UV5-T7 gene 1*) (*DE3*)	[28]
**Plasmids**		
pBAD24	vector containing pBAD promoter	[29]
pEC86	*ccmABCDEFGH gene cluster* inserted into pACYC184	[14]
pTorC-SS	soluble *torC* coding sequence inserted into pBAD24 with *pelB* signal sequence	this study
pTorAD	plasmid containing *torAD* coding sequence inserted into pJF119EH, Amp^r^	[30]
pET-Sp	periplasmic TorS coding sequence cloned into pET21	[7]

## Data Availability

All data are included in the manuscript.

## Supplementary Materials

The following supporting information can be downloaded at https://www.mdpi.com/article/10.3390/ijms252413331/s1.

## References

[1] Baraquet C., Theraulaz L., Guiral M., Lafitte D., Mejean V., Jourlin-Castelli C. (2006). TorT, a member of a new periplasmic binding protein family, triggers induction of the Tor respiratory system upon trimethylamine N-oxide electron-acceptor binding in *Escherichia coli*. J. Biol. Chem..

[2] Jourlin C., Ansaldi M., Mejean V. (1997). Transphosphorylation of the TorR response regulator requires the three phosphorylation sites of the TorS unorthodox sensor in *Escherichia coli*. J. Mol. Biol..

[3] Simon G., Jourlin C., Ansaldi M., Pascal M.C., Chippaux M., Mejean V. (1995). Binding of the TorR regulator to cis-acting direct repeats activates tor operon expression. Mol. Microbiol..

[4] McCrindle S.L., Kappler U., McEwan A.G. (2005). Microbial dimethylsulfoxide and trimethylamine-N-oxide respiration. Adv. Microb. Physiol..

[5] Mejean V., Iobbi-Nivol C., Lepelletier M., Giordano G., Chippaux M., Pascal M.C. (1994). TMAO anaerobic respiration in *Escherichia coli*: Involvement of the tor operon. Mol. Microbiol..

[6] Iobbi-Nivol C., Crooke H., Griffiths L., Grove J., Hussain H., Pommier J., Mejean V., Cole J.A. (1994). A reassessment of the range of c-type cytochromes synthesized by *Escherichia coli* K-12. FEMS Microbiol. Lett..

[7] Gon S., Giudici-Orticoni M.T., Mejean V., Iobbi-Nivol C. (2001). Electron transfer and binding of the c-type cytochrome TorC to the trimethylamine N-oxide reductase in *Escherichia coli*. J. Biol. Chem..

[8] Thony-Meyer L., Fischer F., Kunzler P., Ritz D., Hennecke H. (1995). *Escherichia coli* genes required for cytochrome c maturation. J. Bacteriol..

[9] Gon S., Jourlin-Castelli C., Theraulaz L., Mejean V. (2001). An unsuspected autoregulatory pathway involving apocytochrome TorC and sensor TorS in *Escherichia coli*. Proc. Natl. Acad. Sci. USA.

[10] Gon S., Patte J.C., Mejean V., Iobbi-Nivol C. (2000). The torYZ (yecK bisZ) operon encodes a third respiratory trimethylamine N-oxide reductase in *Escherichia coli*. J. Bacteriol..

[11] Berks B.C., Ferguson S.J., Moir J.W., Richardson D.J. (1995). Enzymes and associated electron transport systems that catalyse the respiratory reduction of nitrogen oxides and oxyanions. Biochim. Biophys. Acta.

[12] Berks B.C., Richardson D.J., Reilly A., Willis A.C., Ferguson S.J. (1995). The napEDABC gene cluster encoding the periplasmic nitrate reductase system of Thiosphaera pantotropha. Biochem. J..

[13] Shaw A.L., Hochkoeppler A., Bonora P., Zannoni D., Hanson G.R., McEwan A.G. (1999). Characterization of DorC from *Rhodobacter capsulatus*, a c-type cytochrome involved in electron transfer to dimethyl sulfoxide reductase. J. Biol. Chem..

[14] Arslan E., Schulz H., Zufferey R., Kunzler P., Thony-Meyer L. (1998). Overproduction of the *Bradyrhizobium japonicum* c-type cytochrome subunits of the cbb3 oxidase in *Escherichia coli*. Biochem. Biophys. Res. Commun..

[15] Meerman H.J., Georgiou G. (1994). High-level production of proteolytically sensitive secreted proteins in *Escherichia coli* strains impaired in the heat-shock response. Ann. N. Y. Acad. Sci..

[16] Genest O., Ilbert M., Mejean V., Iobbi-Nivol C. (2005). TorD, an essential chaperone for TorA molybdoenzyme maturation at high temperature. J. Biol. Chem..

[17] Ansaldi M., Bordi C., Lepelletier M., Mejean V. (1999). TorC apocytochrome negatively autoregulates the trimethylamine N-oxide (TMAO) reductase operon in *Escherichia coli*. Mol. Microbiol..

[18] Simala-Grant J.L., Weiner J.H. (1998). Modulation of the substrate specificity of *Escherichia coli* dimethylsulfoxide reductase. Eur. J. Biochem..

[19] daCosta K.A., Vrbanac J.J., Zeisel S.H. (1990). The measurement of dimethylamine, trimethylamine, and trimethylamine N-oxide using capillary gas chromatography-mass spectrometry. Anal. Biochem..

[20] Schneider F., Lowe J., Huber R., Schindelin H., Kisker C., Knablein J. (1996). Crystal structure of dimethyl sulfoxide reductase from *Rhodobacter capsulatus* at 1.88 A resolution. J. Mol. Biol..

[21] Kaufmann P., Duffus B.R., Mitrova B., Iobbi-Nivol C., Teutloff C., Nimtz M., Jansch L., Wollenberger U., Leimkuhler S. (2018). Modulating the Molybdenum Coordination Sphere of *Escherichia coli* Trimethylamine N-Oxide Reductase. Biochemistry.

[22] Evans R., O’Neill M., Pritzel A., Antropova N., Senior A., Green T., Zidek A., Bates R., Blackwell S., Yim J. (2021). Protein complex prediction with AlphaFold-Multimer. bioRxiv.

[23] Jumper J., Evans R., Pritzel A., Green T., Figurnov M., Ronneberger O., Tunyasuvunakool K., Bates R., Zidek A., Potapenko A. (2021). Highly accurate protein structure prediction with AlphaFold. Nature.

[24] Rodrigues M.L., Scott K.A., Sansom M.S.P., Pereira I.A.C., Archer M. (2008). Quinol Oxidation by c-Type Cytochromes: Structural Characterization of the Menaquinol Binding Site of NrfHA. J. Mol. Biol..

[25] Stahl K., Warneke R., Demann L., Brememkamp R., Hormes B., Brock O., Stülke J., Rappsilber J. (2024). Modelling protein complexes with crosslinking mass spectroscopy and deep learning. Nat. Comm..

[26] Feng C., Kedia R.V., Hazzard J.T., Hurley J.K., Tollin G., Enemark J.H. (2002). Effect of solution viscosity on intramolecular electron transfer in sulfite oxidase. Biochemistry.

[27] Baba T., Ara T., Hasegawa M., Takai Y., Okumura Y., Baba M., Datsenko K.A., Tomita M., Wanner B.L., Mori H. (2006). Construction of Escherichia coli K-12 in-frame, single-gene knockout mutants: The Keio collection. Mol. Syst. Biol..

[28] Studier F.W., Moffatt B.A. (1986). Use of bacteriophage T7 RNA polymerase to direct selective high-level expression of cloned genes. J. Mol. Biol..

[29] Guzman L.M., Belin D., Carson M.J., Beckwith J. (1995). Tight regulation, modulation, and high-level expression by vectors containing the arabinose PBAD promoter. J. Bacteriol..

[30] Pommier J., Mejean V., Giordano G., Iobbi-Nivol C. (1998). TorD, a cytoplasmic chaperone that interacts with the unfolded trimethylamine N-oxide reductase enzyme (TorA) in *Escherichia coli*. J. Biol. Chem..

[31] Tiedemann K., Iobbi-Nivol C., Leimkuhler S. (2022). The Role of the Nucleotides in the Insertion of the bis-Molybdopterin Guanine Dinucleotide Cofactor into apo-Molybdoenzymes. Molecules.

[32] Mueller U., Förster R., Hellmig M., Huschmann F.U., Kastner A., Malecki P., Pühringer S., Röwer M., Sparta K., Steffien M. (2015). The macromolecular crystallography beamlines at BESSY II of the Helmholtz-Zentrum Berlin: Current status and perspectives. Eur. Phyd. J. Plus.

[33] Krug M., Weiss M.S., Heinemann U., Mueller U. (2012). XDSAPP: A graphical user interface for the convenient processing of diffraction data using XDS. J. Appl. Crystallog..

[34] Adams P.D., Afonine P.V., Bunkóczi G., Chen V.B., Davis I.W., Echols N., Headd J.J., Hung L.-W., Kapral G.J., Grosse-Kunstleve R.W. (2010). PHENIX: A comprehensive Python-based system for macromolecular structure solution. Acta Crystallogr. D Biol. Crystallogr..

[35] Emsley P., Lohkamp B., Scott W.G., Cowtan K. (2010). Features and development of Coot. Acta Crystallogr. Sect. D Biol. Crystallogr..

[36] Joosten R.P., Long F., Murshudov G.N., Perrakis A. (2014). The PDB_REDO server for macromolecular structure model optimization. IUCrJ.

[37] Hughes C.S., Moggridge S., Muller T., Sorensen P.H., Morin G.B., Krijgsveld J. (2019). Single-pot, solid-phase-enhanced sample preparation for proteomics experiments. Nat. Protoc..

[38] Rappsilber J., Mann M., Ishihama Y. (2007). Protocol for micro-purification, enrichment, pre-fractionation and storage of peptides for proteomics using StageTips. Nat. Protoc..

[39] John A., Kramer M., Lehmann M., Kunz H.H., Aarabi F., Alseekh S., Fernie A., Sommer F., Schroda M., Zimmer D. (2024). Degradation of FATTY ACID EXPORT PROTEIN1 by RHOMBOID-LIKE PROTEASE11 contributes to cold tolerance in Arabidopsis. Plant Cell.

[40] Cox J., Mann M. (2008). MaxQuant enables high peptide identification rates, individualized p.p.b.-range mass accuracies and proteome-wide protein quantification. Nat. Biotechnol..

[41] Yilmaz S., Busch F., Nagaraj N., Cox J. (2022). Accurate and Automated High-Coverage Identification of Chemically Cross-Linked Peptides with MaxLynx. Anal. Chem..

[42] Abramson J., Adler J., Dunger J., Evans R., Green T., Pritzel A., Ronneberger O., Willmore L., Ballard A.J., Bambrick J. (2024). Accurate structure prediction of biomolecular interactions with AlphaFold3. Nature.

[43] Pettersen E.F., Goddard T.D., Huang C.C., Meng E.C., Couch G.S., Croll T.I., Morris J.H., Ferrin T.E. (2021). UCSF ChimeraX: Structure Visualization for Researchers, Educators, and Developers. Nat. Comm..

