# Supplementary material for: Purification and Electron Transfer from Soluble c-Type Cytochrome TorC to TorA for Trimethylamine N-Oxide Reduction
Source: Int J Mol Sci. 2024 Dec 12;25(24):13331. doi: 10.3390/ijms252413331 (PMC11727998; doi:10.3390/ijms252413331)
Supplement: Supplementary file 1 [file ijms-25-13331-s001.zip › ijms-3358574-supplementary.pdf]

Supporting information

Purification and Electron Transfer from Soluble c-Type Cytochrome TorC to TorA for Trimethylamine N-Oxide Reduction

Alka Panwar [1], Berta M. Martins [2], Frederik Sommer [3], Michael Schroda [3], Holger Dobbek [2], Chantal Iobbi-Nivol [4], Cécile Jourlin-Castelli [4] and Silke Leimkühler [1,*]

[1] Department of Molecular Enzymology, Institute of Biochemistry and Biology, University of Potsdam, Karl-Liebknecht Str. 24-25, 14476 Potsdam, Germany; panwaralka2016@gmail.com

[2] Department of Biology, Humboldt-Universität zu Berlin, Unter den Linden, 10999 Berlin, Germany; berta.martins@hu-berlin.de (B.M.M.); holger.dobbek@biologie.hu-berlin.de (H.D.)

[3] Molekulare Biotechnologie & Systembiologie, RPTU Kaiserslautern-Landau, Paul-Ehrlich Straße 23, 67663 Kaiserslautern, Germany; frsommer@rptu.de (F.S.); m.schroda@rptu.de (M.S.)

[4] CNRS, BIP, Aix-Marseille University, 13005 Marseille, France; iobbi@imm.cnrs.fr (C.I.-N.); jourlin@imm.cnrs.fr (C.J.-C.)

* Correspondence: sleim@uni-potsdam.de

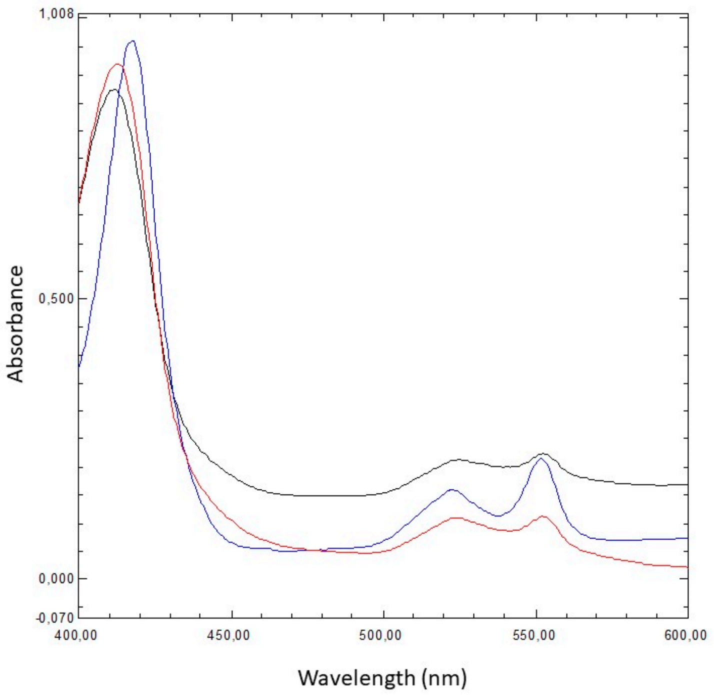

Fig S1: UV-Vis spectra of purified soluble TorC. Visible spectrum of soluble TorC (10 µM) in the oxidized state (red line) and after reduction with ascorbate (0.1mM) (black line) and with dithionite (0.1mM) (blue line).

A) TorA:TorC

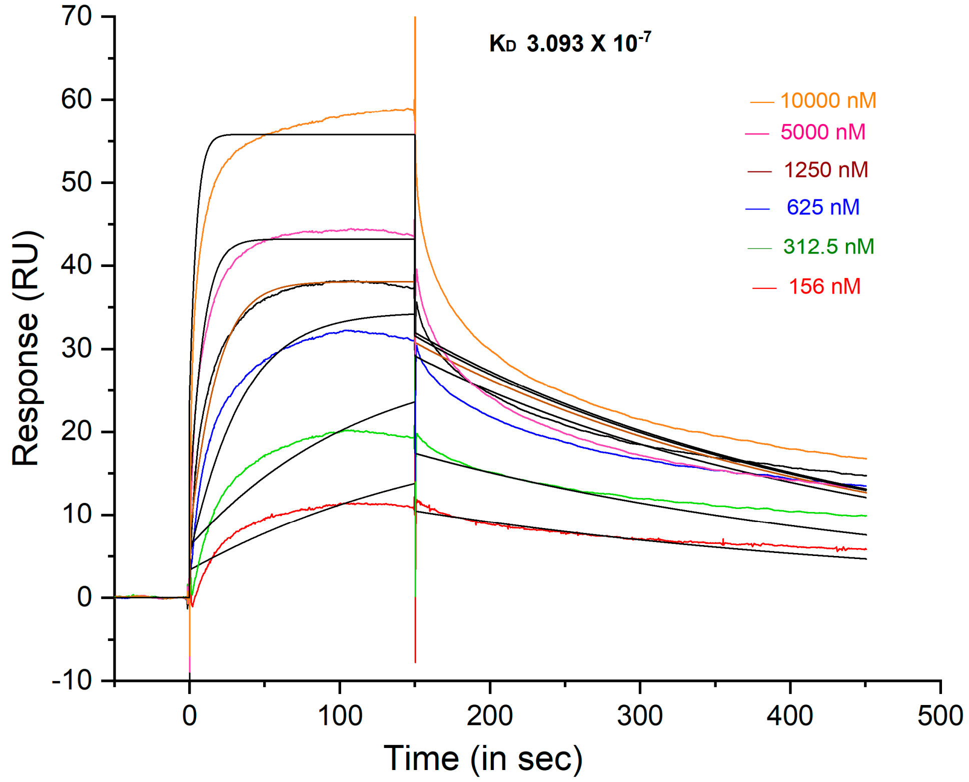

B) apoTorA:TorC

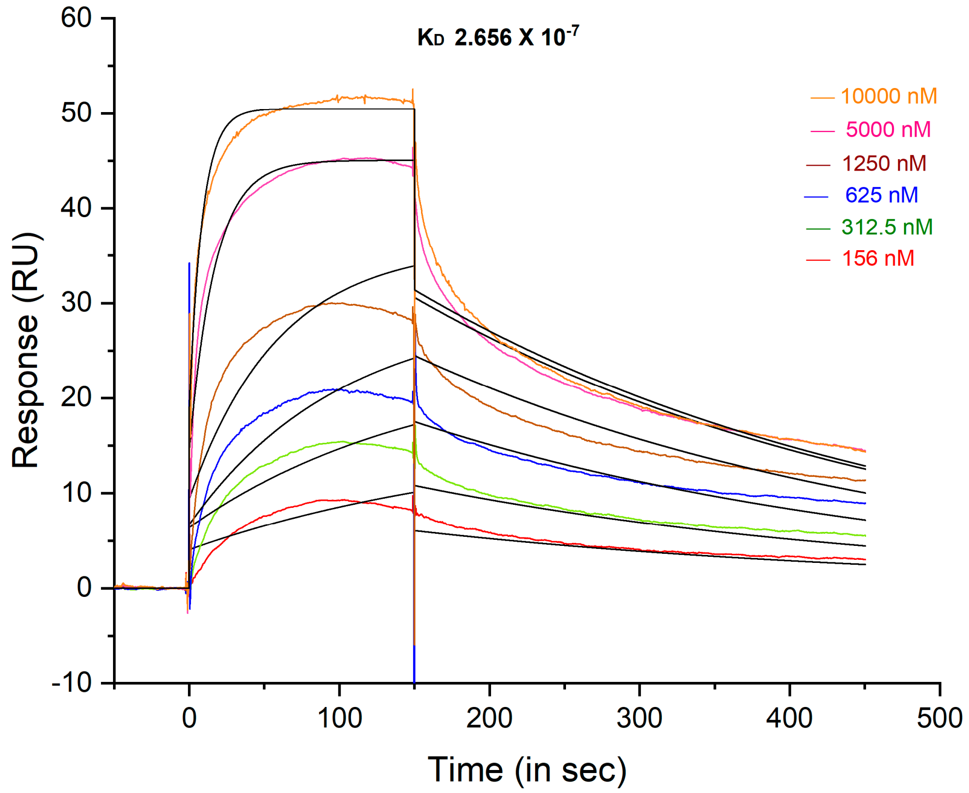

C) TorS:holoTorC

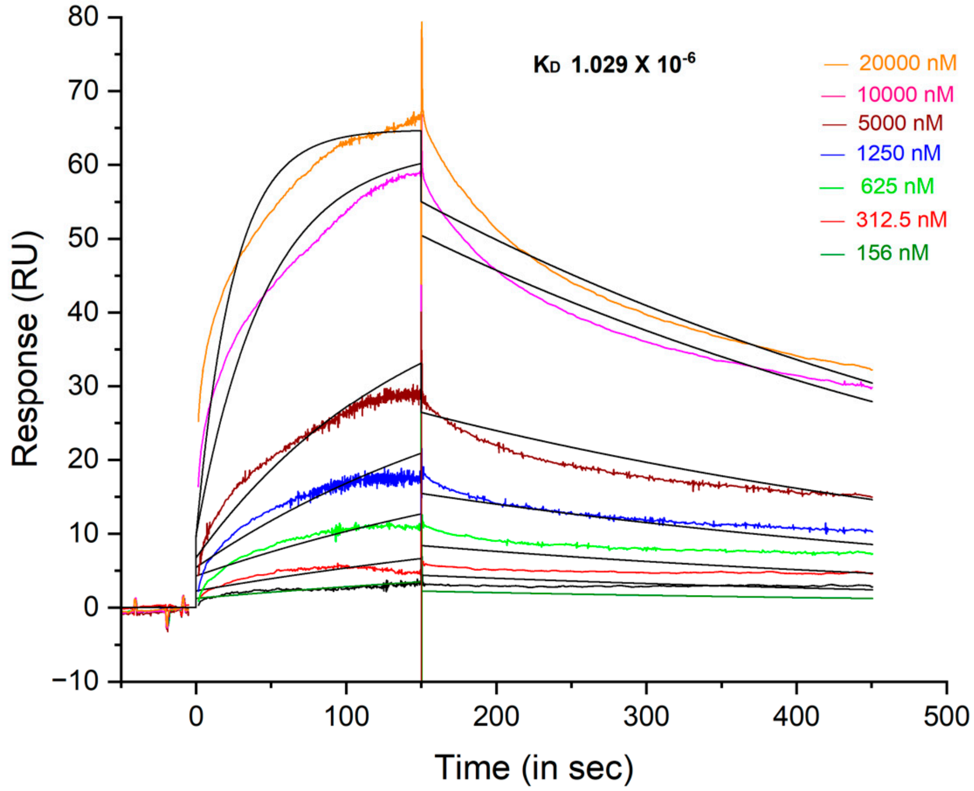

D) TorS:apoTorC

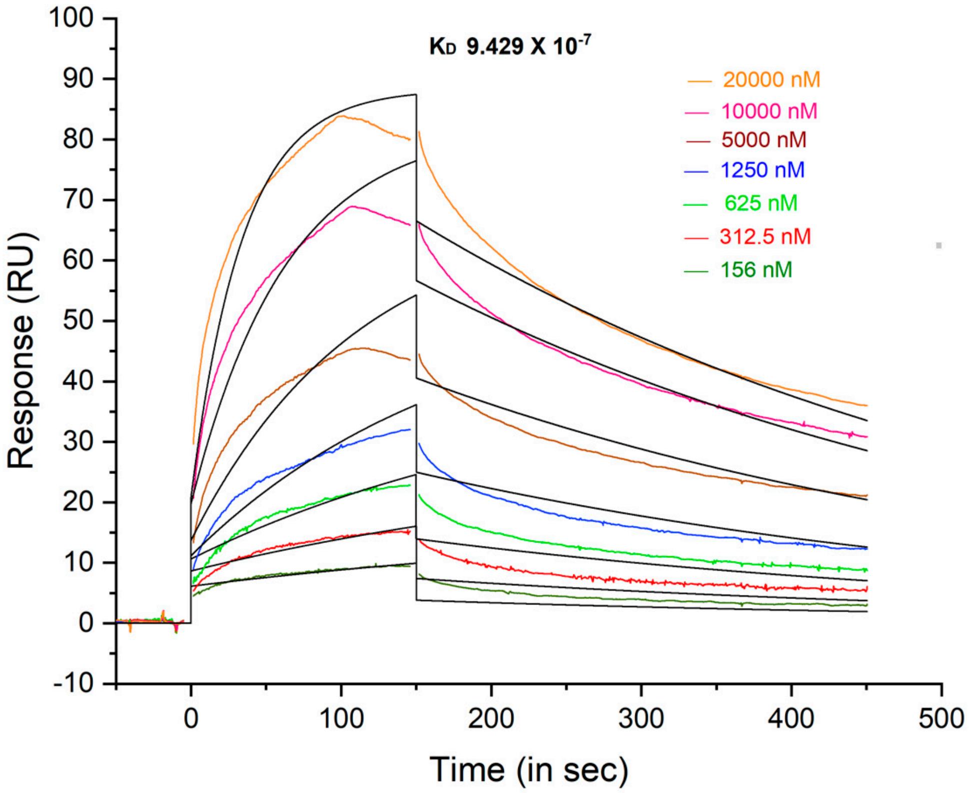

Figure S2: SPR analysis using Biacore system. A) TorA (immobilized) with TorC, B) apoTorA (immobilized) with TorC , C) TorS (immobilized) with holoTorC and, D) TorS(immobilized) with apoTorC

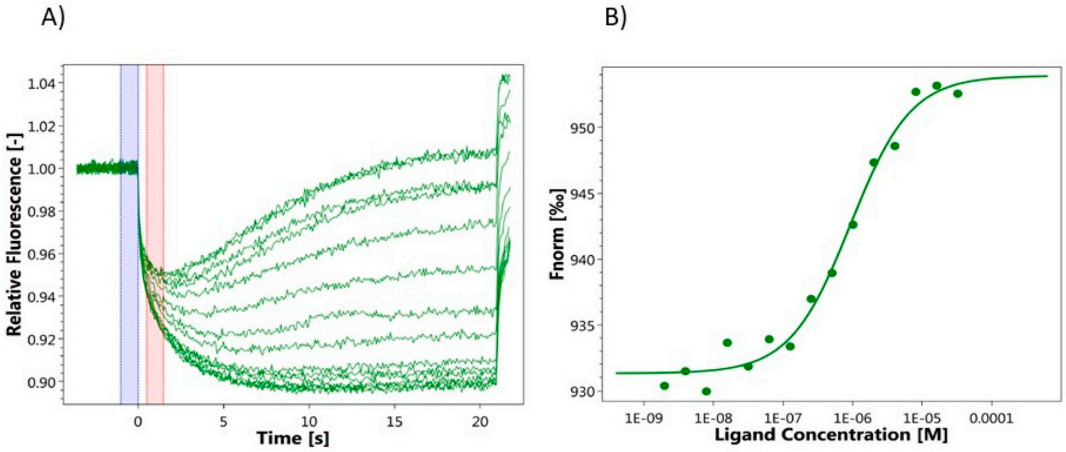

Fig S3: **MST analysis of the interaction of TorA with TorC.** A) The binding experiment (MST traces) showing the changes in thermophoretic movement upon binding of fluorescent TorA (5 nM) with different concentrations of non-fluorescent TorC (stock concentration 65 µM). The blue region indicates the cold spot and the red region shows the hot spot during the thermophoresis. B) The dose-response curve obtained for TorA-TorC interaction shows the plot of Fnorm against the ligand concentration.

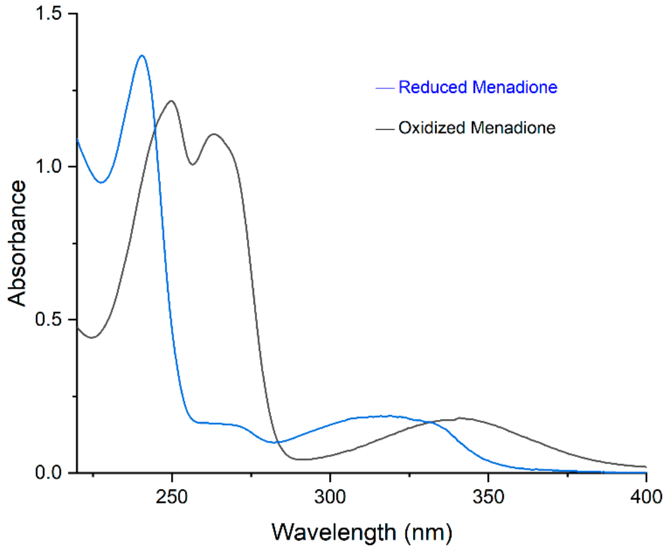

Figure S4: UV-Vis spectra of 50 µM oxidized menadione (black line) and 50 µM reduced menadione with 100 µM sodium borohydride (blue line) in 20 mM phosphate buffer, pH 7.4. Spectra were recorded between 200 nm and 400 nm. The reduced form of menadione has an absorption maximum at 240 nm while the oxidized form has an absorption maximum at 260 nm (Extinction coefficient at 260 nm=17.18 $mM^{-1}cm^{-1}$) [20].

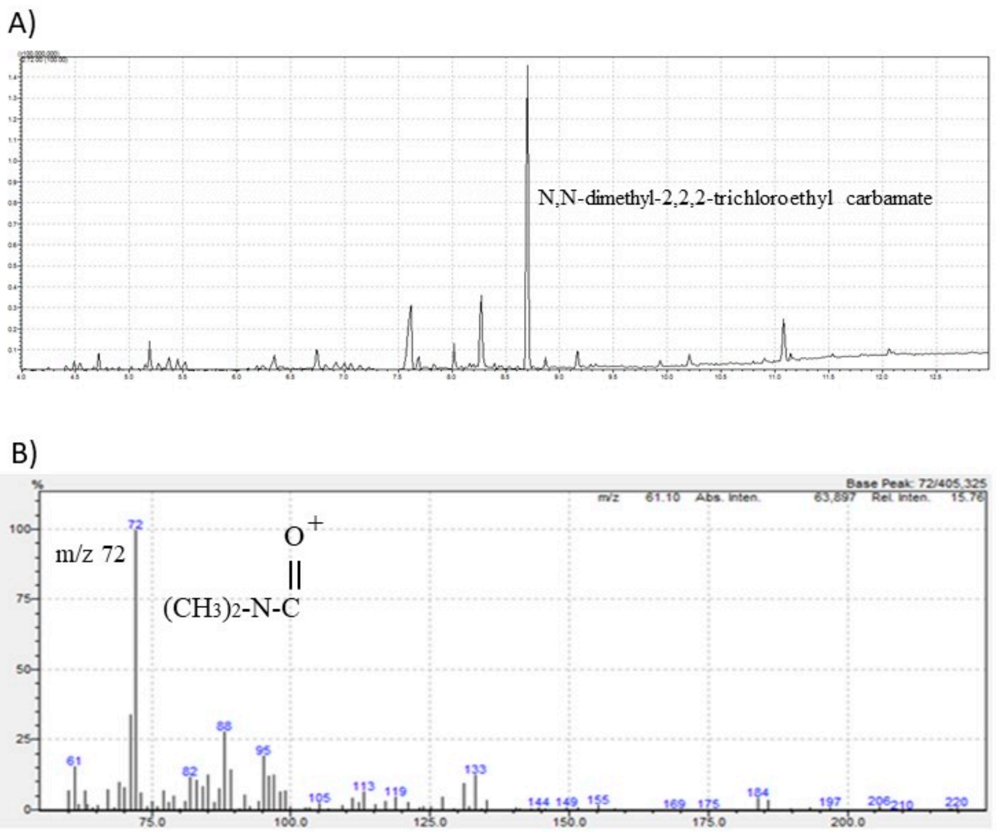

Fig S5: Detection of the derivatized form of TMA using GC-MS. A) GC-MS chromatogram produced by the derivatized form of the TMA (N,N-dimethyl-2,2,2-trichloroethyl carbamate). B) Mass spectra observed from the major peak in (A), showing the relative abundance of ion fragment m/z 72 originating from derivatized TMA.

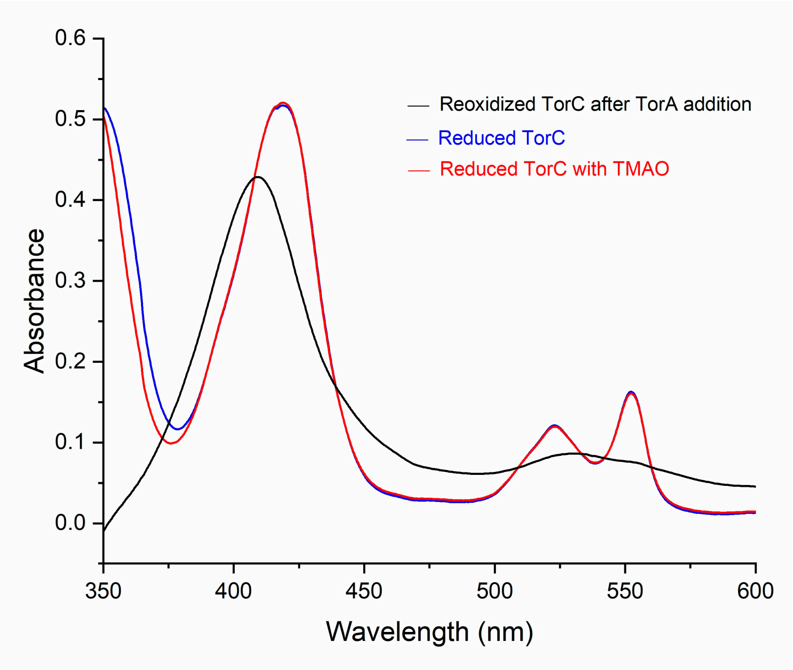

Fig S6: UV-Vis spectra of soluble TorC. TorC (10 µM) reduced by menadiol (in blue,), after addition of TMAO (50 mM, in red) and reoxidized soluble TorC after the subsequent addition of TorA (10 µM) (in black).

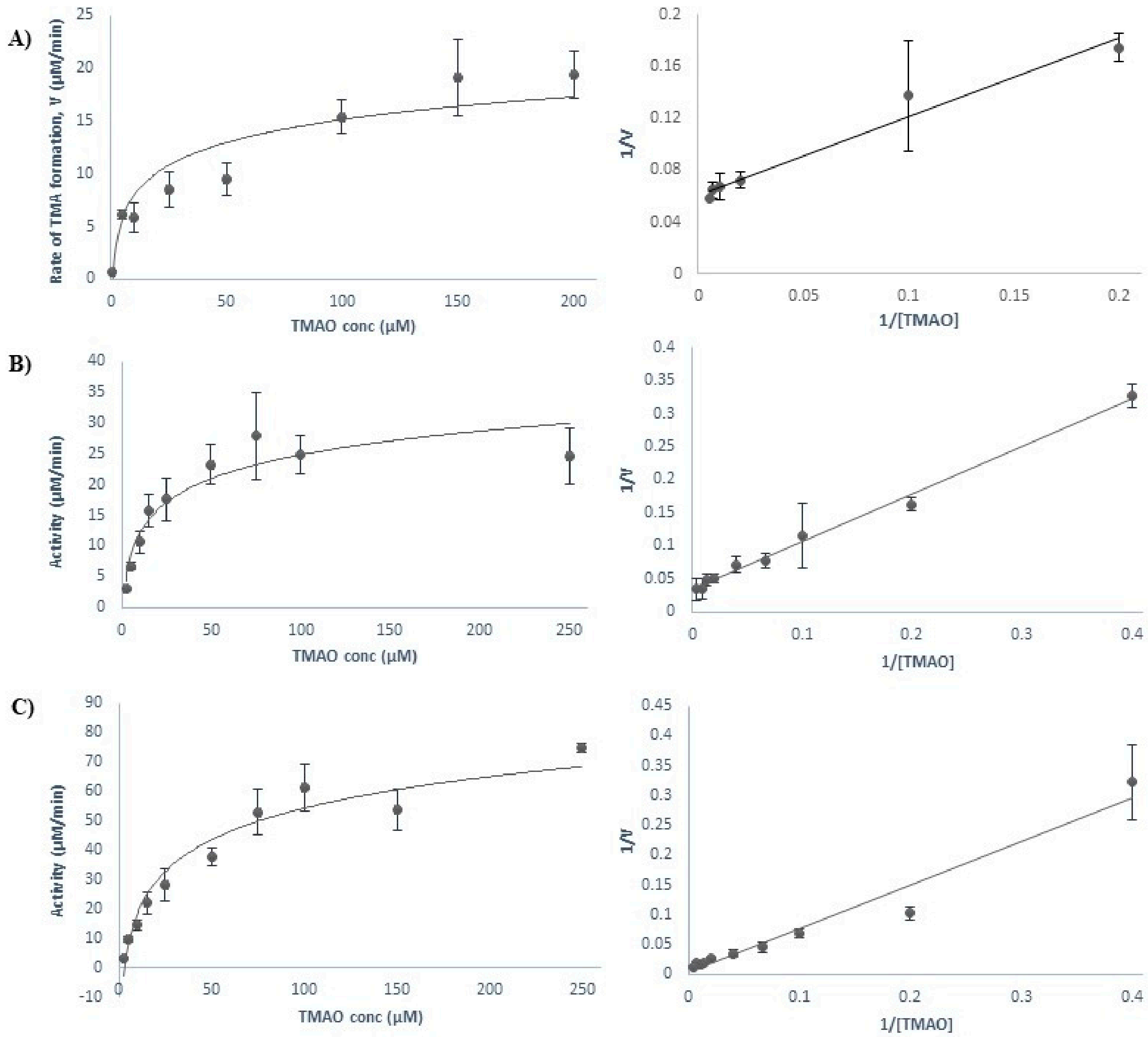

Fig S7: Dependence of rate of TMA formation against substrate concentrations with reduced menadione (A), reduced benzyl viologen (B) and reduced methyl viologen as electron sources (C). The left panel shows Michaelis Menten plots of the reaction rates against different TMAO concentrations in the assay. The right panel shows the Lineweaver- Burk plots of the same data.

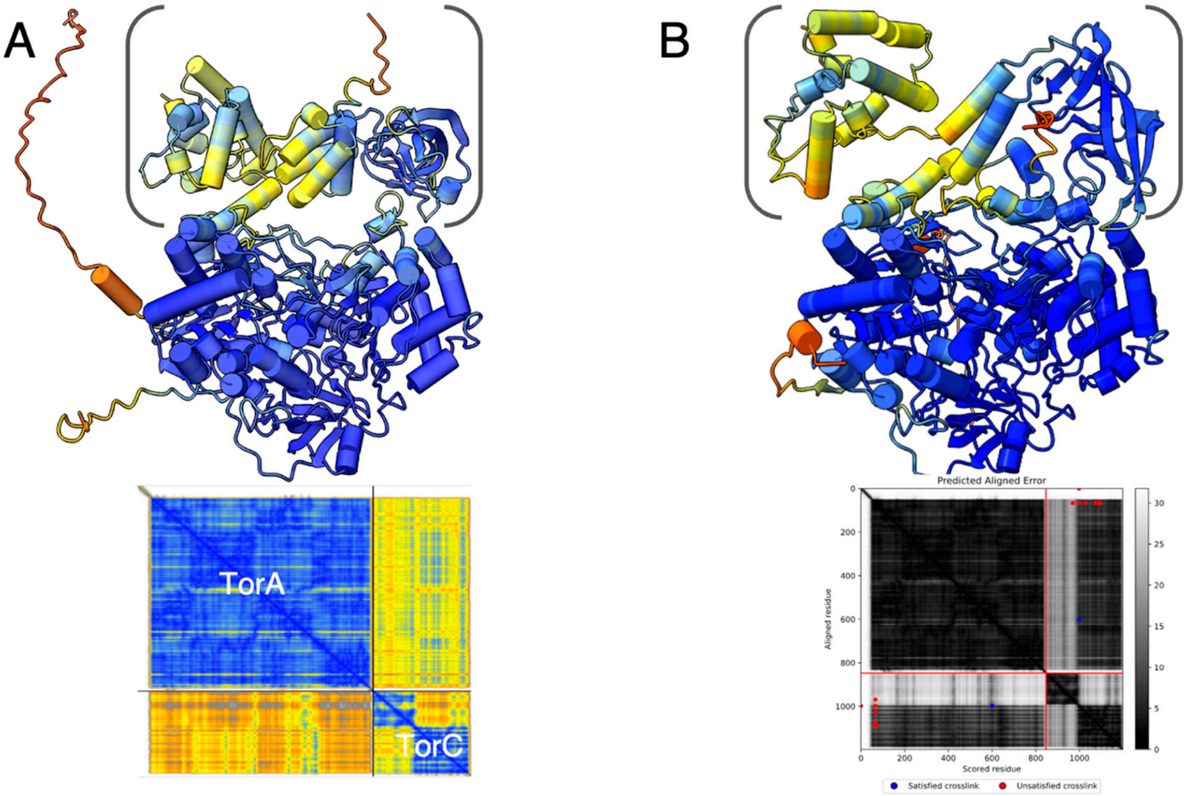

Fig S8: AI-models of TorA-TorC complexes depicted as cartoons and colored by their predicted local distance difference test (pLDDT), and respective predicted aligned error (PAE) diagrams. TorC is shown within the brackets. The color scheme represents the model confidence based on the pLDDT value: dark blue for very high (pLDDT > 90), light blue for high (90 > pLDDT > 70), yellow for low (70 > pLDDT > 50), and orange for very low (pLDDT < 50. **A)** generated by AlphaFold-multimer. The PAE file was visualized in ChimeraX. **B)** generated by AlphaLink2. The PAE file was automatically generated by the program as implemented in CoLab.

Table S1: Crystallographic data collection and refinement statistics for TorA

	TorA (PDB-ID 9H4T)
**Data collection**	
**Source**	BL 14.1 BESSY
**Wavelength (Å)**	0.9184
**Resolution range (Å)**	47.79 – 1.86 (1.98-1.86)
**Space group**	P 1 21 1
**Cell constant (a,b,c (Å), α,β,γ (°))**	93.66 117.47 100.05 90 103.25 90
**Total, Unique reflections**	389176 (61348), 159613 (25104)
**Multiplicity**	2.4 (2.4)
**Rmeas (%)**	11.4 (69.7)
**$RCC_{1/2}$ (%)**	99.4 (60.9)
**Completeness (%)**	90.88 (88.70)
**I/sigma(I)**	7.45 (1.50)
**Wilson B-factor**	22.09
**Refinement**	
**Reflections used in refinement**	159577 (10001)
**Reflections used for R-free**	2099 (132)
**Model R_{work} / R_{free} factors**	0.2351 / 0.2888
**Ramachandran statistics (%)**	
**Favored/allowed/disfavored**	96.77 / 3.23 / 0.00
**RMS-deviation from ideal geometry**	
**Bonds (Å)**	0.010
**Angles (°)**	1.28
**Coordinate error (Å)[a]**	0.16
**Rotamer outliers (%)**	1.44
**Clashscore**	7.11
**Average B-factor ($Å^2$)**	28.44
**macromolecules**	27.33
**ligands**	39.14
**solvent**	34.19

Values in parentheses are given for the highest resolution shell. [a] maximum-likelihood based estimated coordination error.
